# Performance-Enhanced Activated Carbon Electrodes for Supercapacitors Combining Both Graphene-Modified Current Collectors and Graphene Conductive Additive

**DOI:** 10.3390/ma11050799

**Published:** 2018-05-15

**Authors:** Rubing Wang, Yuting Qian, Weiwei Li, Shoupu Zhu, Fengkui Liu, Yufen Guo, Mingliang Chen, Qi Li, Liwei Liu

**Affiliations:** 1 Key Laboratory of Nanodevices and Applications, Suzhou Institute of Nano-Tech and Nano-Bionics, Chinese Academy of Sciences, Suzhou 215123, China; rbwang2013@sinano.ac.cn (R.W.); ytqian2016@sinano.ac.cn (Y.Q.); wwli2009@sinano.ac.cn (W.L.); spzhu2017@sinano.ac.cn (S.Z.); fkliu2015@sinano.ac.cn (F.L.); yfguo2009@sinano.ac.cn (Y.G.); mlchen2011@sinano.ac.cn (M.C.); 2 School of Nano Technology and Nano Bionics, University of Science and Technology of China, Hefei 230026, China; 3 SZGraphene Nanotechnology Co., Ltd., Suzhou 215123, China

**Keywords:** activated carbon, supercapacitor, electrodes, graphene, nickel foam, current collector, conductive additive, electrochemical properties

## Abstract

Graphene has been widely used in the active material, conductive agent, binder or current collector for supercapacitors, due to its large specific surface area, high conductivity, and electron mobility. However, works simultaneously employing graphene as conductive agent and current collector were rarely reported. Here, we report improved activated carbon (AC) electrodes (AC@G@NiF/G) simultaneously combining chemical vapor deposition (CVD) graphene-modified nickel foams (NiF/Gs) current collectors and high quality few-layer graphene conductive additive instead of carbon black (CB). The synergistic effect of NiF/Gs and graphene additive makes the performances of AC@G@NiF/G electrodes superior to those of electrodes with CB or with nickel foam current collectors. The performances of AC@G@NiF/G electrodes show that for the few-layer graphene addition exists an optimum value around 5 wt %, rather than a larger addition of graphene, works out better. A symmetric supercapacitor assembled by AC@G@NiF/G electrodes exhibits excellent cycling stability. We attribute improved performances to graphene-enhanced conductivity of electrode materials and NiF/Gs with 3D graphene conductive network and lower oxidation, largely improving the electrical contact between active materials and current collectors.

## 1. Introduction

With the increasing requirement of energy storage devices in the modern technology society, supercapacitors have drawn great attention due to the higher power density, quicker charge and discharge rate, and longer cycle life compared with traditional lithium-ion batteries [1,2,3,4]. According to the different charge storage modes, supercapacitors can be divided into two kinds, the pseudocapacitor derived from the reversible redox reaction of active electrode material and electrical double-layer capacitor (EDLC) depending on electrostatic ion attraction and accumulation on the electrode/electrolyte interface [5,6,7].

At present, the relatively low energy density is still a key bottleneck of supercapacitors. The formula $E=\frac{1}{2}{\mathrm{CV}}^{2}$, where E is the energy density, C is the mass specific capacitance, and V is the potential range, illustrates that energy density is closely related to the specific capacitance and potential range [8,9,10,11]. As a result, improving specific capacitance or potential range is the fundamental approach to achieve higher energy density. Now, most researches concentrate on selecting appropriate active materials and electrolytes to get higher specific capacitances and broader working voltages. Carbon materials (graphene, carbon nanotube, AC et al.) with high conductivity, nanostructure transition metal oxide (MnO_2_, Co_3_O_4_, Ni_X_Mn_1-X_O et al.) with high capacitance and their composites are the most famous active materials being studied now [12,13,14,15,16,17]. Furthermore, asymmetric supercapacitors have also been fabricated to obtain higher energy densities through broadening working voltages [18,19]. However, there are many other factors on equivalent series resistance (ESR) easily to be neglected, such as current collectors and conductive additives, which often influence performance enhancement by decreasing the resistances of the active materials, current collectors, the contact resistances between them, and so on [20,21,22,23].

Current collector, as an essential part of a supercapacitor, plays an important role in collecting current and supporting electrode materials. The conductivity and adhesion with active materials of a current collector can directly influence the specific capacitance [24,25,26,27]. Nickel is one of the most popular current collectors of supercapacitors owing to its good conductivity, mechanical properties, stability in aqueous alkali and strong adhesions with active materials [28,29,30]. To further improve the above properties is still the main research orientation in modifying nickel current collectors. Many works about modifying nickel current collectors by chemical or electrochemical etches have been published to increase the surface roughness in order to strengthen the adhesion with active materials [31,32]. In addition, methods like jet electrodepositing were reported to prepare a multi-pore nickel to increase the surface roughness [33]. However, these methods are always involved in the usage of alkali or acid solutions, and even relatively complex fabrication processes, which are environmentally unfriendly, lack safety, and are difficult in industry promotion. And then a kind of modified nickel foam (NiF) current collectors with intrinsic high specific surface area, covered by few layer graphene through chemical vapor deposition (CVD) has been reported [21,34]. These graphene-modified NiFs can not only increase the conductivity of the current collectors but also strengthen the combination between active materials and current collectors, markedly improving the electrical contacts of active materials and current collector. In addition, the existence of the CVD graphene layers protects the NiFs from the erosion of oxygen and water in the air, which has been rarely published.

Moreover, conductive additives also are of great importance in an operational electrode. The most common conductive additive is carbon black (CB) [35]. The additive amounts of CB are always up to 10 wt % of the total electrode weight, even more in some poor conductivity systems, which makes the proportion of active material reduced, as well as the overall specific capacitance. Efficient conductive additives are needed to achieve high performance electrodes with lower additive amounts. Highly conductive graphene is a potential candidate for conductive additives because of its well-known excellent properties, such as high conductivity, specific surface area, light weight, and so on [36,37].

Here, activated carbon (AC) electrodes were prepared with CVD graphene-modified nickel foams (NiF/Gs) as current collectors and high quality few-layer graphene as conductive additive (AC@G@NiF/G). On one hand, few-layer graphene as conductive additive directly improves the conductivity of the electrodes. On the other hand, CVD graphene on NiFs not only increases the conductivity and stability of the current collector, but also as a buffer layer to strengthen the adhesion to active material, providing a highly effective electrical contact for fast charge transfer from AC active material to current collectors with the underlying 3D graphene network [21]. Due to the synergistic effect of NiF/G current collectors and few-layer graphene conductive additive, the electrochemical properties of as-prepared AC@G@NiF/G electrodes are higher than those of AC electrodes with CB conductive additive (AC@CB@NiF/G) and common NiF current collectors free of graphene (AC@G@NiF). Just at low weight of graphene conductive additive of 5 wt %, the AC@G@NiF/G (denoted as AC@G@NiF/G-5) electrode shows highest electrochemical properties (specific capacitance 123.6 F/g and energy density 17.2 Wh/kg at the current density of 1 A/g), which are higher than those of AC@CB@NiF/G electrode with 10 wt % CB (AC@CB@NiF/G-10) (96 F/g and 13.3 Wh/kg) and AC@G@NiF electrode with 5 wt % graphene (AC@G@NiF-5) (78.3 F/g and 10.9 Wh/kg). However, the property reduce at higher additive amount can be attributed to the planar structure of graphene sheets, which hinder the electrolyte ion transport in electrode material [38]. A symmetric supercapacitor (SSA) fabricated with AC@G@NiF/G-5 electrodes shows excellent cycling stability without any decrease of specific capacitance after 10,000 cycles. This work can be recognized with reference value for researchers for fundamental research and industrial application of graphene-modified supercapacitors or batteries.

## 2. Materials and Methods

### 2.1. Materials

The activated carbon (AC) powder (Kuraray YF-50) was received from Kuraray international trade CO., LTD. (Shanghai, China). The graphene powder (GRF-H-FLGA-01, lateral size 5–10 μm, thickness ~2 nm) was brought from SZgraphene nanotechnology Company (Suzhou, China). The Potassium hydroxide (KOH, GR, ≥85 wt %) and N-Methyl pyrrolidone (NMP, AR, ≥99.0 wt %) were brought from Sinopharm Chemical Reagent Company (Shanghai, China). The nickel foam (NiF, HGP, 110 ppi, 500 g/m^2^) was obtained from HGP technology CO., LTD (Wuzhou, China). The aqueous membrane (NKK-MPF 30AC-100), Polyvinylidene Fluoride (PVDF, Solvay TA-6020/1001) and carbon black (CB, Ketjenblack EC300J, Lion CO., Tokyo, Japan) were purchased on Taobao (https://item.taobao.com/item.htm?spm=a230r.1.14.16.73d46067nVvMYK&id=530857358840&ns=1&abbucket=3#detail).

### 2.2. CVD Growth of G/NiF Current Collector

NiFs were placed in the middle of a quartz tube furnace, and then the furnace should be vacuumized and filled with Ar twice to eliminate the residual air in the furnace. Before growth NiFs were heated to 1000 °C in 30 min under the gas flow of Ar (200 sccm) and H_2_ (200 sccm), and then annealed at 1000 °C for 10 min under the gas flow of Ar (200 sccm) and H_2_ (300 sccm), in order to remove the oxide layers on NiFs. Methane (CH_4_) was then injected into the quartz as carbon source to growth graphene under ambient pressure with gas flow of Ar (400 sccm), H_2_ (200 sccm) and CH_4_ (100 sccm) at 1000 °C. After 10 min, the quartz was fast cooled to room temperature under the gas flow of Ar (200 sccm) and H_2_ (300 sccm). After growth of graphene, the color of NiF/Gs changed from shallow to deep.

### 2.3. Preparation of AC Electrodes and Assembly of Supercapacitors

Certain amount of AC, graphene, CB and PVDF were mixed in NMP by a high-shear dispersion homogenizer (U400/80-220, BW Motor Co., Shanghai, China) at 1800 rpm/min for 4 h to obtain uniformly dispersed slurries. And then, these slurries were dip coated on NiF/G or NiF current collectors and dried at 130 °C in vacuum for 24 h to prepare AC electrodes. The current collectors were made into two specifications (square with 1 cm × 1 cm and circular piece with diameter 13 mm). The square electrodes were used to take electrochemical measurements with a standard three-electrode method. The circular electrodes were used to fabricate symmetrical supercapacitors. Two electrodes with similar mass of electrode materials were assembled into a button capacitor with the membrane (NKK-MPF 30AC-100, Kawasaki, Japan), electrolyte (6 M KOH) and button cell module (CR 2025).

### 2.4. Characterizations and Electrochemical Measurements

The morphology of NiF/G current collector, raw material powders and AC electrodes was observed using scanning electron microscope (SEM, Quanta FEG 250, FEI Co., Hillsboro, OR, USA). The Raman spectra were carried out by a Raman spectrometer (LabRam HR800-UV-NIR, HORIBA Jobin Yvon, Paris, France, λ = 532.15 nm). The X-ray photoelectron spectroscopy (XPS) spectra were measured by an X-ray photoelectron spectroscopy (Thermo Scientific ESCALAB 250 XI, Waltham, MA, USA). The transmission electron microscopy (TEM, Tecnai G2 F20 S-Twin, FEI Co.) was used to characterize the morphology and layer number of high quality few-layer graphene. The specific surface areas were tested by a specific surface area analyzer (BET) (Micromeritics TriStar II 3020, Micromeritics instrument (Shanghai) LTD., Shanghai, China). The surface resistance of electrode materials was measured by a surface resistance meter (RC2175, EDTM Co., New York, NY, USA). The test of resistivity and resistance of current collectors were carried on by a multifunction digital four-probe tester (ST 2258C, Suzhou Jingge Electronics Co., Suzhou, China). All the electrochemical measurements of electrodes and supercapacitors were performed by an electrochemical workstation (CHI660D, CH Instruments, Inc., Shanghai, China) in 6 M KOH aqueous solution as the electrolyte, using a standard three-electrode method and two-electrode method, respectively.

## 3. Results and Discussion

**Figure 1:**
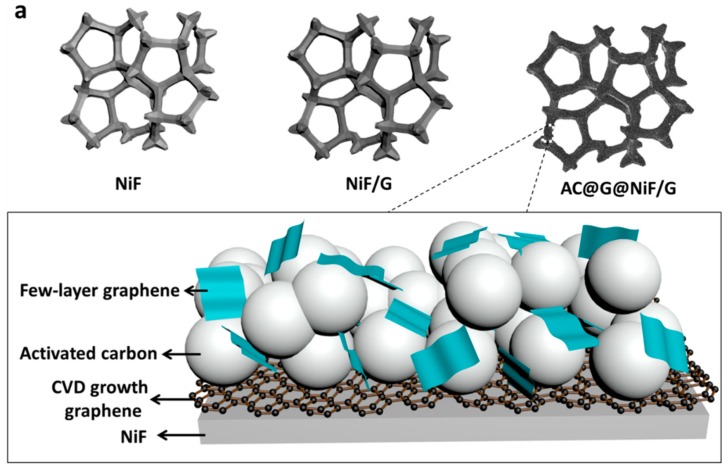

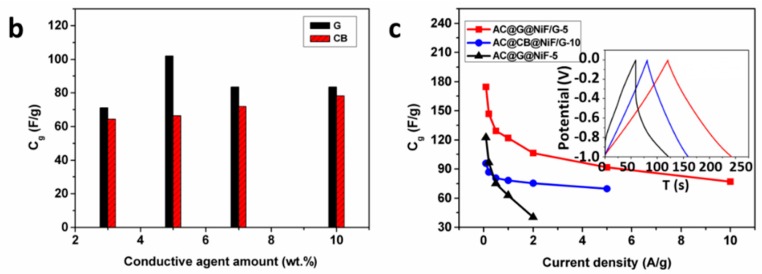
The improved AC@G@NiF/G electrodes. (**a**) Schematic illustration for the AC@G@NiF/G electrode using graphene-modified NiF as current collector and few-layer graphene sheets as conductive additive. (**b**) The specific capacitances of AC@G@NiF/G and AC@CB@NiF/G electrodes with different conductive additive amounts of 3 wt %, 5 wt %, 7 wt %, and 10 wt % at the scan rate of 50 mV/s. (**c**) The specific capacitances of AC@G@NiF/G-5, AC@CB@NiF/G-10, and AC@G@NiF electrodes, as functions of the current densities (the inset shows the corresponding GCD curves at the current density of 1 A/g, and the black, blue, and red curves represent AC@G@NiF-5, AC@CB@NiF/G-10, and AC@G@NiF/G-5, respectively).

AC@G@NiF/G electrodes with different graphene conductive additive amounts of 3–10 wt % were prepared using NiF/Gs as current collectors and few-layer graphene sheets as conductive additive (Figure 1a). The AC@G@NiF/G electrodes show enhanced performances compared with both electrodes on NiF current collectors and electrodes with common CB as conductive additive, especially in capacitance and rate capability (Figure 1b,c). Figure 1b shows that the specific capacitance of AC@G@NiF/G electrode just with 5 wt % graphene has exceeded that of AC@CB@NiF/G electrode with 10 wt % CB, indicating few-layer graphene is potential conductive to realize higher performance at lower additive amount, instead of common CB.

### 3.1. The Roles of NiF/G Current Collectors

The AC@G@NiF/G electrodes show the higher specific capacitances, energy densities and rate capabilities, which can hardly occur without the use of NiF/G current collectors. Graphene can be obviously observed on the surface of NiF after CVD growth, which remarkably increases the roughness of the current collector (Figure S1a–c). And electrode materials are well adhered to the NiF/G current collectors (Figure S1e–h). Through the Raman spectrum of 3D graphene after liquid etching NiF/G to remove NiF substrate, CVD graphene is supposed with low defects (Figure S1d).

In order to illuminate the contribution of the NiF/G current collectors, AC electrodes on common NiF current collectors with graphene conductive additive of 5 wt % (AC@G@NiF-5) were made as comparison to AC@G@NiF/G-5 electrodes. Cyclic voltammetry (CV) curves at scan rates of 10–500 mV/s and galvanostatic charge-discharge (GCD) curves at current densities of 0.1–1 A/g within a voltage range of −1–0 V of the AC@G@NiF-5 electrode are shown in Figure S2a,b. Combined with the corresponding specific capacitances as functions of scan rates and current densities (Figure S2c–f), it can be confirmed that electrodes on NiF/G have higher specific capacitances and much better rate capabilities than those on NiF. At the current density of 1 A/g, the electrode on NiF/G gets the higher specific capacitance and energy density (specific capacitance 123.6 F/g and energy density 17.2 Wh/kg) than those of electrode on NiF (specific capacitance 78.3 F/g and energy density 10.9 Wh/kg). According to the CV curves at the scan rate of 50 mV/s in Figure S2e, the shape of CV curve of AC@G@NiF/G-5 electrode is more symmetrical and close to an ideal rectangle, which is equivalent to better reversibility of charge-discharge reaction and capacitance characteristic.

**Figure 2:**
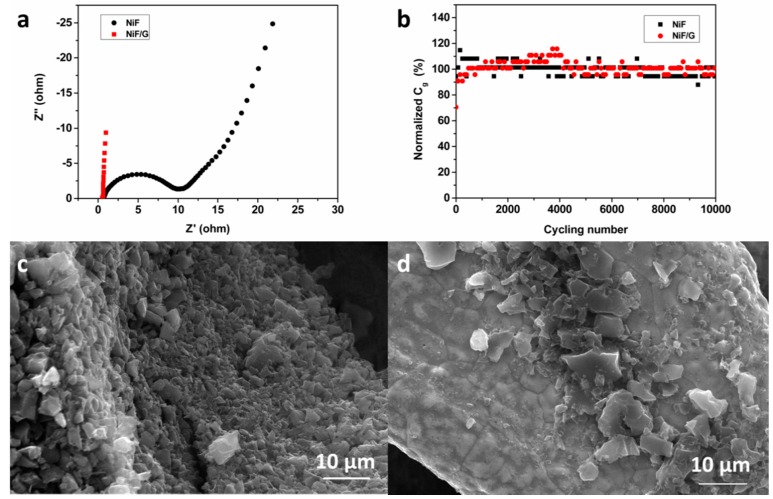
The AC@G@NiF/G-5 electrode and AC@G@NiF-5 electrode. (**a**) The Nyquist plots. (**b**) The capacitance retention of 10,000 cycles. (**c**,**d**) SEM images of AC@G@NiF/G-5 electrode and AC@G@NiF-5 electrode, respectively (scale bar = 10 μm).

This superior rate capability can be attributed to the significantly lowered ESR, which may be derived from the enhanced electrical contact between active materials and current collectors by CVD growth graphene, as evidenced by the electrochemical impedance spectroscopy (EIS) analysis (Figure 2a). The much smaller semicircle in high frequency represents the lower charge-transfer resistance occurring at the electrolyte/electrode interface. The more inclined line in the low frequency of electrode on NiF/G indicates the better modified Warburg impedance related to the diffusion impedance. Good cycling stability is an important target in performance enhancement of supercapacitor electrodes. As demonstrated in Figure 2d, the AC@G@NiF/G-5 electrode exhibits a more stable cycle life and has almost no degradation in specific capacitance after 10,000 charge-discharge cycles.

**Figure 3:**
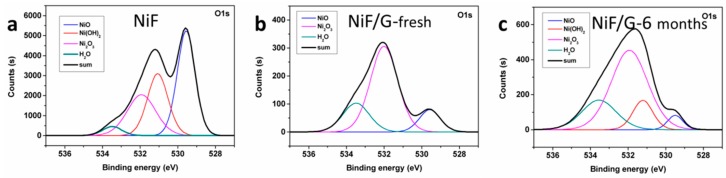
The X-ray photoelectron spectroscopy (XPS) spectra of NiF and NiF/G. The fitting O1s XPS spectra of (**a**) NiF, (**b**) NiF/G-fresh (just prepared several hours before), and (**c**) NiF/G-6 months (placing in air for six months).

The roles of NiF/G current collectors are considered as three aspects. Firstly, graphene of high specific surface area depositing directly on NiF as a carbon buffer layer between AC material and NiF, strengthens the adhesion of active material on current collector, and then improves the contact at interface. It is consistent with Figure 2c,d, which demonstrates the SEM images of AC electrodes with 5 wt % graphene additive on NiF/G and NiF current collectors. The active material of the electrode on NiF/G has a more sufficient adhesion on the current collector than that on NiF. Secondly, after CVD growth of graphene, highly conductive 3D graphene network on top of NiF frame enhances the conductivity of current collector, which enhances the electrical contact between AC electrode materials and NiF/G current collectors and then facilitates the charge transfer [21]. Thirdly, the CVD growth graphene also protects the NiF substrates from corrosion of oxygen and water in air. As a result, NiF/Gs have lower surface oxidation and better chemical stability, which is beneficial to the further cycle life. As proved by the X-ray photoelectron spectroscopy (XPS) spectra of O1s in Figure 3. Natural oxide and hydroxide layer will be formed on the surface of NiF due to the oxygen and water in the air, corresponding to NiO at 529.56 eV, Ni_2_O_3_ at 531.93 eV, and Ni(OH)_2_ at 531.07 eV in the O1s spectrum of NiF (Figure 3a). Peaks at 533.48 eV are thought of the adherent H_2_O from air (Figure 3a–c). After the process of CVD growth graphene, the height of H_2_O peaks of NiF/Gs obviously decrease and slightly rise up over time of six months (Figure 3b,c), denoting the good water blocking character of graphene cover layer. The weaken NiO peak and absent Ni(OH)_2_ peak of the fresh NiF/G in Figure 3b mean that oxidation and hydroxylation of nickel are suppressed obviously with the introduction of CVD graphene. And over six months, NiO and Ni(OH)_2_ only appear slightly. The reduction of oxide and hydroxide layer will further improve the electrical contact between active materials and current collectors. It is worth noting that the CVD process not only grows the graphene cover layer but also removes the oxide and hydroxide layer on the top of NiF, which reduces resistivity (from 0.38 mΩ∙cm of NiF to 0.358 mΩ∙cm of NiF/G).

All these three points about the graphene buffer layer have a high conductivity 3D network and chemical stability which guarantees the superior electrochemical performance of the AC@G@NiF/G-5 electrode, such as higher specific capacitance, energy density, better rate capability, cycling stability, and lower resistance.

### 3.2. The Effect of Graphene Conductive Additive

**Figure 4:**
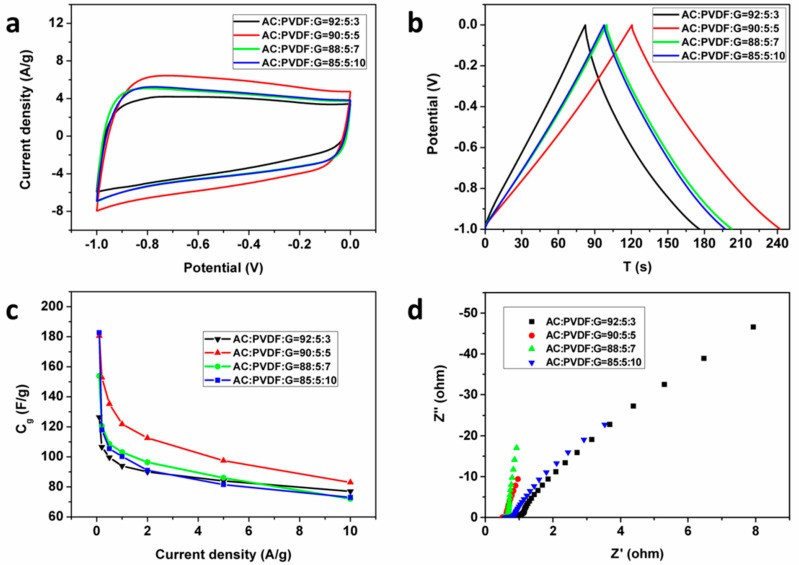
The electrical properties of AC@G@NiF/G electrodes with different graphene conductive additive amounts from 3 to 10 wt %. (**a**) CV curves at the scan rate of 50 mV/s. (**b**) GCD curves at the current density of 1 A/g. (**c**) The corresponding mass specific capacitances as functions of current densities. (**d**) The Nyquist plots. The legends in figures represent the different AC@G@NiF/G electrodes with different contents of electrode material components (active material AC, binder PVDF, and conductive additive graphene).

The electrochemical properties of AC@G@NiF/G electrodes with graphene conductive additive varying from 3 wt % to 10 wt % are demonstrated in Figure 4. CV curves at the scan rate of 50 mV/s and GCD curves at the current density of 1 A/g within a voltage range from −1 to 0 V were measured to evaluate the mass specific capacitance of AC@G@NiF/G electrodes (Figure 2a,b). It was found that with the increase of graphene conductive additive, the electrochemical properties get improved first, which can be attributed to highly conductive graphene improving electrical microenvironment in AC active material of the composite electrodes, facilitating electrolyte ion diffusion and charge transfer in AC, and then promoting the value of specific capacitances. While beyond the graphene additive amount of 5 wt %, specific capacitance values show slight decrease. Because the planar structure of graphene sheets will tend to block the transport of electrolyte ions in electrode materials and impede the transfer of charge. It can be proved by the Nyquist plots in Figure 4d. The high-frequency intercept on the real axis in the Nyquist plots represents ohmic resistance, including the ionic resistance of electrolyte, the contact resistance between active material and current collector, and the intrinsic resistance of active material. The result clearly reflects that the addition of graphene effectively reduces contact resistances between AC materials and NiF/G current collectors and the intrinsic resistance of AC electrode materials for these AC@G@NiF/G electrodes. Moreover, the slopes in low frequency region also reflect the Warburg impedance, related to the electrolyte ion diffusion in electrodes, indicating that the ion diffusion is enhanced first and then reduced with the increase of graphene. The highest specific capacitance was observed in the AC@G@NiF/G-5 electrode, which was confirmed with both the specific capacitance 102.0 F/g at the scan rate of 50 mV/s and 123.6 F/g at the current density of 1 A/g. The similar law is also obtained in the rate capabilities (Figure 4c), corresponding to the mass specific capacitances of AC@G@NiF/G electrodes as functions of current densities. The similar trend is also found in the BET specific surface areas of AC electrode materials. With the graphene additive amount increasing from 3 wt % to 10 wt %, the BET specific surface area increases first, and then decreases (Figures S3 and S4). But the peak of BET specific surface area is at 7 wt % rather than 5 wt % as the peak of electrochemical properties. It can be supposed that the planar structure of graphene sheets will block the electrolyte ion transport in electrode materials [38]. Although graphene additive helps enhance the BET specific surface area, when graphene conductive additive amount is beyond 5 wt %, we inferred the block effect does more than specific surface area.

Few-layer graphene nanosheets were used as conductive additive instead of traditional CB to prepare the AC@G@NiF/G composite electrodes, which plays an important role in performance enhancement of electrodes. As a contrast, AC electrodes on NiF/G current collectors with CB as conductive additive (AC@CB@NiF/G electrodes) were made by the same method as AC@G@NiF/G electrodes. Figures S5–S7 show the Raman spectrum, morphology and surface resistance of AC@G@NiF/G and AC@CB@NiF/G electrodes, which reveals that electrode with graphene as conductive agent has higher conductivity than that of CB under the same additive amount. It can be attributed to the high quality of few-layer graphene (5–10 layers), which is also proved by the characterizations of the graphene raw material (Figure S8).

**Figure 5:**
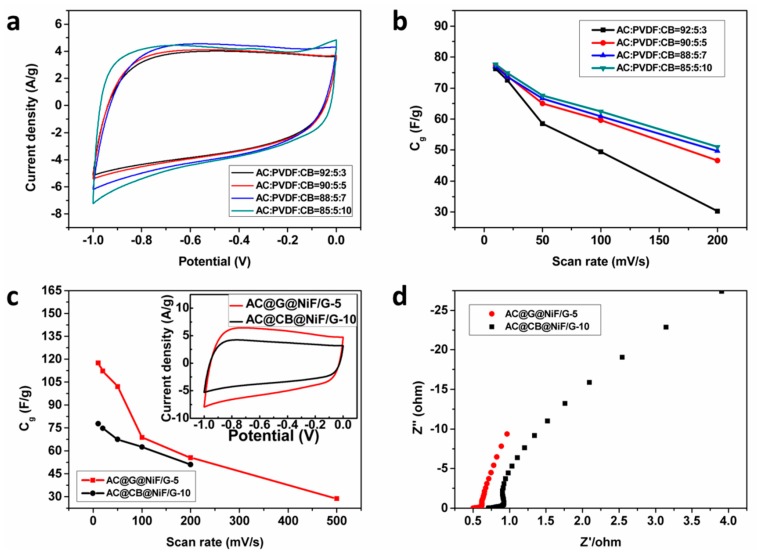
Comparisons of AC@G@NiF/G and AC@CB@NiF/G electrodes. (**a**) CV curves at the scan rate of 50 mV/s, and (**b**) the corresponding mass specific capacitances as functions of scan rates of AC@CB@NiF/G electrodes with different CB amounts. The legends in (**a**) and (**b**) represent the different AC@G@NiF/G electrodes with different contents of electrode material components (active material AC, binder PVDF, and conductive additive graphene). (**c**) The mass specific capacitances as functions of scan rates (The inset shows the CV curves at the scan rate of 50 mV/s), and (**d**) the Nyquist plots of AC@G@NiF/G-5 and AC@CB@NiF/G-10 electrodes.

The CV curves at the scan rate of 50 mV/s and corresponding specific capacitances as functions on scan rates of AC@CB@NiF/G electrodes with different CB additive amount of 3–10 wt % are demonstrated in Figure 5a,b, which indicates that specific capacitances of AC@CB@NiF/G electrodes are improved with the increase of CB conductive additive. The AC@CB@NiF/G-10 electrode has the highest specific capacitance and best rate capability among electrodes with CB. However, it is yet inferior to the AC@G@NiF/G-5 electrode, both in specific capacitances and rate capabilities (Figure S9 and Figure 5c,d). It can be seen from the CV and GCD curves of AC@CB@NiF/G-10 electrode (Figure S10) and those of AC@G@NiF/G-5 electrode (Figure S9a,c). This is because the usage of graphene conductive additive makes the electrode with better conductivity, improves the electrical contact at the electrolyte/electrode interface and facilitates the electrolyte ion diffusion and charge transport.

Table 1 lists some previous works and our graphene/AC composite electrodes for supercapacitors. Our electrode displays much better cycling stability than other graphene/AC composite electrodes and possesses the capacitance commensurate to current level.

### 3.3. The Symmetric Supercapacitor Assembled by AC@G@NiF/G-5 Electrodes

**Figure 6:**
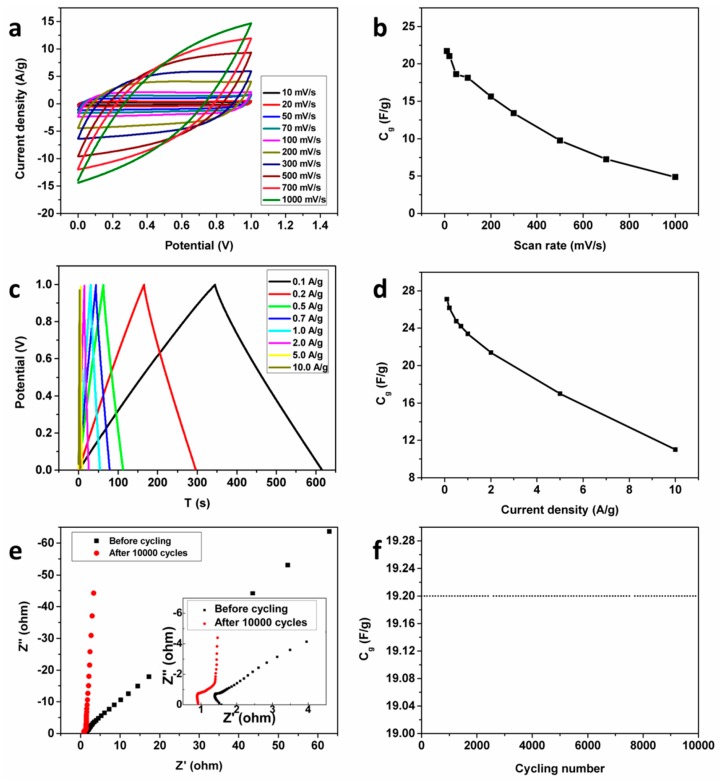
The electrochemical properties of the symmetric supercapacitor assembled by AC@G@NiF/G-5 electrodes. (**a**) CV curves at scan rates of 10–1000 mV/s. (**b**) The corresponding mass specific capacitances as a function of scan rates. (**c**) GCD curves at different current densities of 0.1–10 A/g. (**d**) The corresponding mass specific capacitances as a function of current densities of the SSC assembled by AC@G@NiF/G-5 electrodes. (**e**) The Nyquist plots before and after cycling for 10,000 times (The inset is the enlarged image of high frequency region). (**f**) The capacitance retention of 10,000 cycles.

Two AC@G@NiF/G-5 electrodes with the same mass of the active material were assembled to a symmetric supercapacitor (SSC) with the electrolyte being a 6 M KOH aqueous solution. The CV curves Raman 10–500 mV/s shown in Figure 6a exhibit quasi-rectangular shapes, even at a high scan rate of 500 mV/s, suggesting good reversibility and typical electrical double-layer capacitive characteristics of our device. The GCD curves at various current densities of 0.1–10 A/g in the potential range of 0–1 V are plotted in Figure 6c. The quasi-equicrural triangle curves also indicate a good capacitive behavior for the supercapacitor. Excellent cycling stability is demonstrated by Figure 6f displaying almost no decrease of capacitance after 10,000 cycles. Furthermore, the Nyquist plots of the SSC before and after cycling in Figure 6e demonstrate that a little bigger curvature of semicircle and left shift of intersection of the horizontal axis in the high frequency region, which reveal the almost unchanged ESR after cycling. The slop larger than 1 in the low frequency region reflects the well held capacitive behavior after cycling, which can be attributed to the further sufficient immersion of electrolyte after cycling.

## 4. Conclusions

In summary, we demonstrated a kind of performance-enhanced electrodes of supercapacitors involved in NiF/G current collectors and graphene as the conductive additive. Firstly, the NiF/G current collectors guarantee the conductivity and stability of the current collectors and strengthen the adhesion with active materials, which provide a much more effective electrical contact for fast charge transfer from active materials to current collectors. Secondly, the addition of graphene as the conductive additive directly improves the conductivity of the electrode. Both the NiF/G current collectors and the highly conductive graphene additive make the performances of as-prepared AC@G@NiF/G electrodes superior to those of AC@CB@NiF/G and AC@G@NiF electrodes. Just at low addition of graphene conductive additive of 5 wt %, the AC@G@NiF/G-5 electrodes show best electrochemical properties, specific capacitance 123.6 F/g and energy density 17.2 Wh/kg at the current density of 1 A/g. The SSA assembled by AC@G@NiF/G-5 electrodes also shows excellent cycling stability with almost no decrease of specific capacitance after 10,000 cycles. The results are expected with reference value for researchers for fundamental research and industrial application of graphene-modified supercapacitors or even batteries.

## Figures and Tables

**Table 1:** Electrochemical performances of graphene/AC composite electrodes [39].

Electrode	Role of Graphene	Electrolyte	Specific Capacitance	Capacity Retention Ratio	Energy Density (Wh/kg)	Power Density (W/kg)
Graphene/AC [36]	component of active material	6M KOH	210 F/g (1 mV/s)	94.7% (5000)	22.3	33.2
Graphene/AC [40]	component of active material	KOH	122 F/g	90% (3000)	6.1	-
Graphene/AC fiber [41]	dispersant & binder	1M H_2_SO_4_	43.8 F/g	90.4% (10,000)	-	-
Graphene/AC [42]	component of active material	6M KOH	297 F/g (0.1 A/g)	90% (6000)	6.12	4660
Nitrogen-doped graphene/AC [43]	component of active material	6M KOH	145 F/g (20 mV/s)	98.4% (5000)	-	-
AC@G@NiF/G [our work]	Conductive agent & modification of current collectors	6M KOH	123.6 F/g (1 A/g)	>95% (10,000)	17.2	507.5

## Supplementary Materials

The following are available online at http://www.mdpi.com/1996-1944/11/5/799/s1.
