# Supplementary material for: Performance-Enhanced Activated Carbon Electrodes for Supercapacitors Combining Both Graphene-Modified Current Collectors and Graphene Conductive Additive
Source: Materials (Basel). 2018 May 15;11(5):799. doi: 10.3390/ma11050799 (PMC5978176; doi:10.3390/ma11050799)
Supplement: Supplementary file 1 [file materials-11-00799-s001.pdf]

materials

MDPI

Supplementary

Performance-Enhanced Activated Carbon Electrodes for Supercapacitors Combining Both Graphene-Modified Current Collectors and Graphene Conductive Additive

Rubing Wang [1,2], Yuting Qian [1], Weiwei Li [1,3], Shoupu Zhu [1], Fengkui Liu [1], Yufen Guo [1,3], Mingliang Chen [1], Qi Li [1,*] and Liwei Liu [1,3,*]

[1] Key Laboratory of Nanodevices and Applications, Suzhou Institute of Nano-Tech and Nano-Bionics, Chinese Academy of Sciences, Suzhou 215123, China; rbwang2013@sinano.ac.cn (R.W.); ytqian2016@sinano.ac.cn (Y.Q.); wwli2009@sinano.ac.cn (W.L.); spzhu2017@sinano.ac.cn (S.Z.); fkliu2015@sinano.ac.cn (F.L.); yfguo2009@sinano.ac.cn (Y.G.); mlchen2011@sinano.ac.cn (M.C.)

[2] School of Nano Technology and Nano Bionics, University of Science and Technology of China, Hefei 230026, China

[3] SZGraphene Nanotechnology Co., Ltd., Suzhou 215123, China

* Correspondence: qli2013@sinano.ac.cn (Q.L.); lwliu2007@sinano.ac.cn (L.L.); Tel.: +86-512-62872734

Received: 17 April 2018; Accepted: 9 May 2018; Published: 15 May 2018

Abstract: Graphene has been widely used in the active material, conductive agent, binder or current collector for supercapacitors, due to its large specific surface area, high conductivity, and electron mobility. However, works simultaneously employing graphene as conductive agent and current collector were rarely reported. Here, we report improved activated carbon (AC) electrodes (AC@G@NiF/G) simultaneously combining chemical vapor deposition (CVD) graphene-modified nickel foams (NiF/Gs) current collectors and high quality few-layer graphene conductive additive instead of carbon black (CB). The synergistic effect of NiF/Gs and graphene additive makes the performances of AC@G@NiF/G electrodes superior to those of electrodes with CB or with nickel foam current collectors. The performances of AC@G@NiF/G electrodes show that for the few-layer graphene addition exists an optimum value around 5 wt %, rather than a larger addition of graphene, works out better. A symmetric supercapacitor assembled by AC@G@NiF/G electrodes exhibits excellent cycling stability. We attribute improved performances to graphene-enhanced conductivity of electrode materials and NiF/Gs with 3D graphene conductive network and lower oxidation, largely improving the electrical contact between active materials and current collectors.

Keywords: activated carbon; supercapacitor; electrodes; graphene; nickel foam; current collector; conductive additive; electrochemical properties

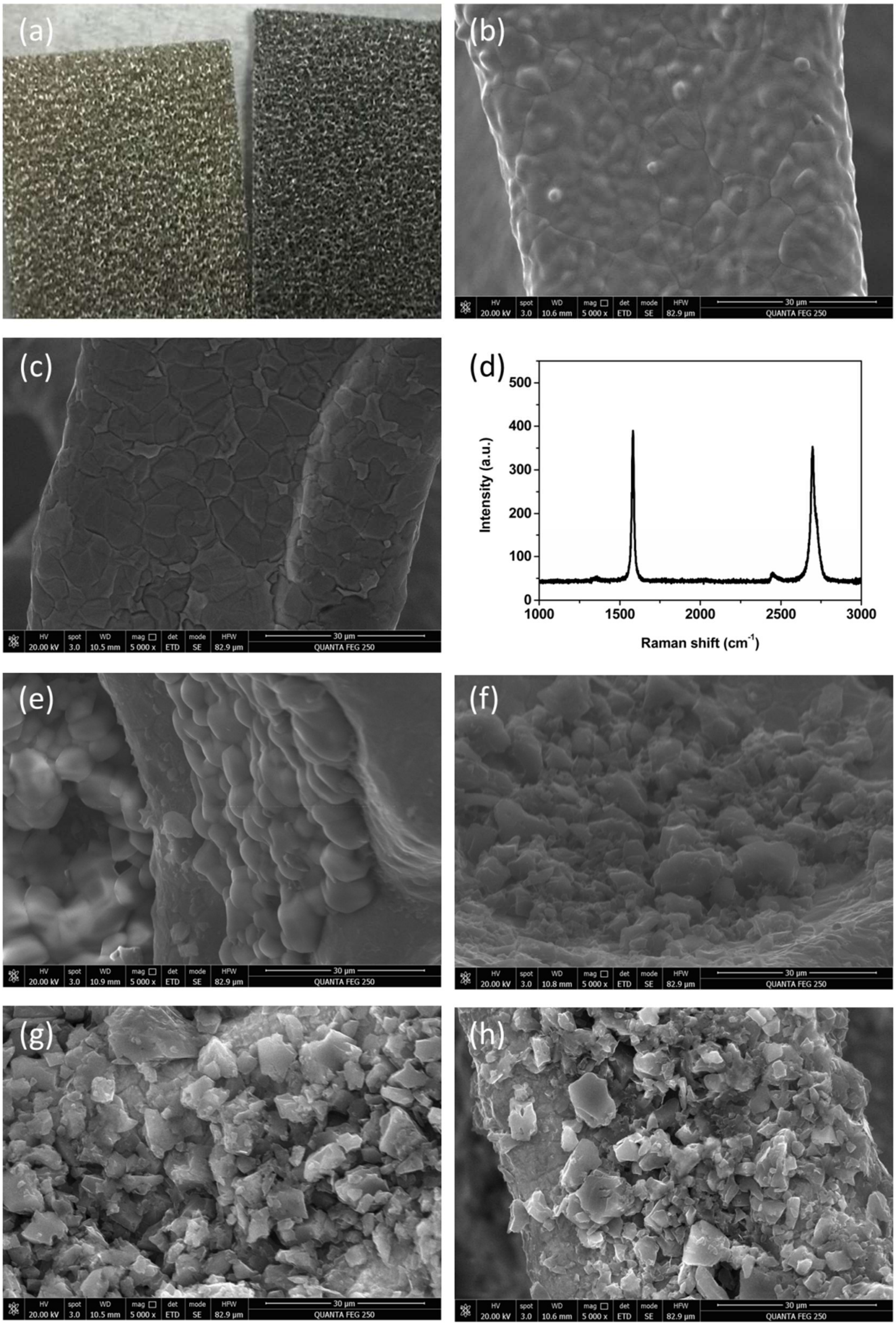

Figure S1. (**a**) The digital image and the typical SEM images of of (**b**) NiF and (**c**) NiF/G current collectors. (**d**) Raman spectrum of 3D graphene frame after the liquid etching of the NiF substrate. SEM images of AC@G@NiF/G electrodes with graphene conductive additive amounts of (**e**) 3 wt %, (**f**) 5 wt %, (**g**) 7 wt % and (**h**) 10 wt %.

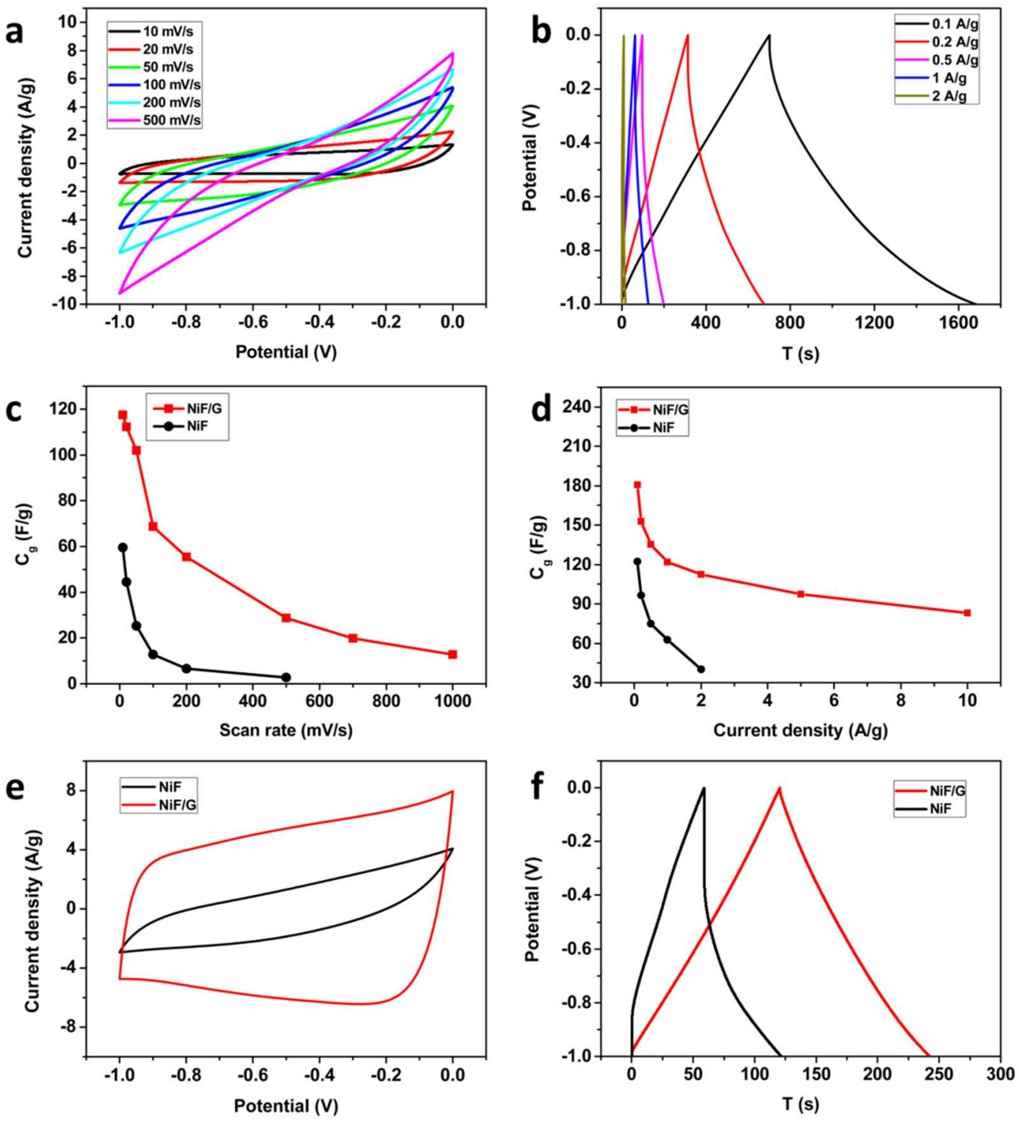

Figure S2. The electrochemical properties of the AC@G@NiF-5 electrode. (**a**) CV curves at scan rate various from 10 to 500 mV/s. (**b**) GCD curves at different current densities from 0.1 to 2 A/g. Mass specific capacitances as functions of (**c**) scan rates and (**d**) current densities. (**e**,**f**) are the corresponding CV curves at the scan rate of 50 mV/s and GCD curves at the current density of 1 A/g, respectively.

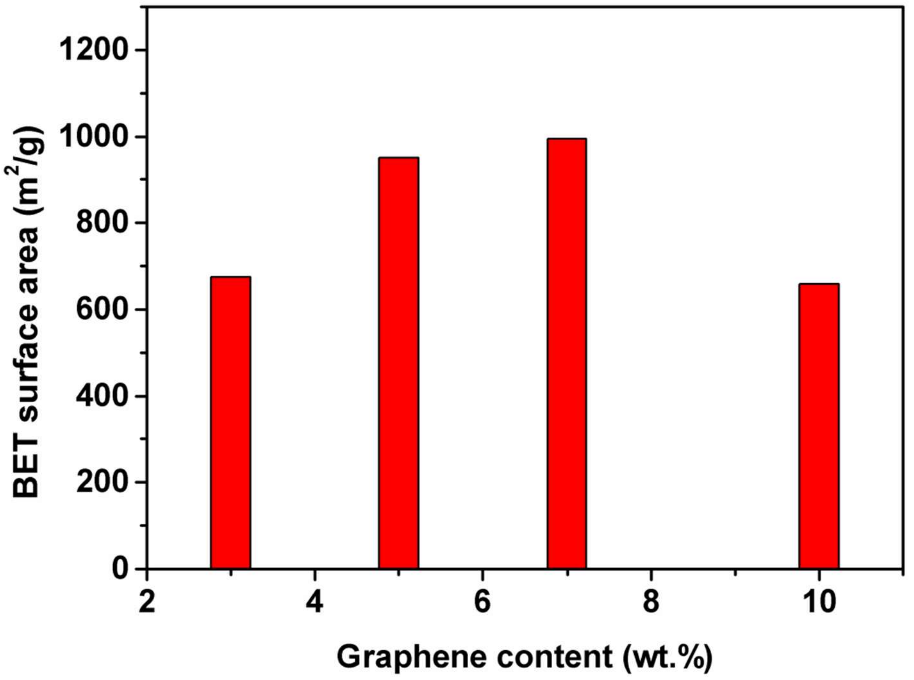

Figure S3. The BET surface areas of AC/graphene/PVDF electrode materials with different graphene conductive additive amounts of 3, 5, 7 and 10 wt %.

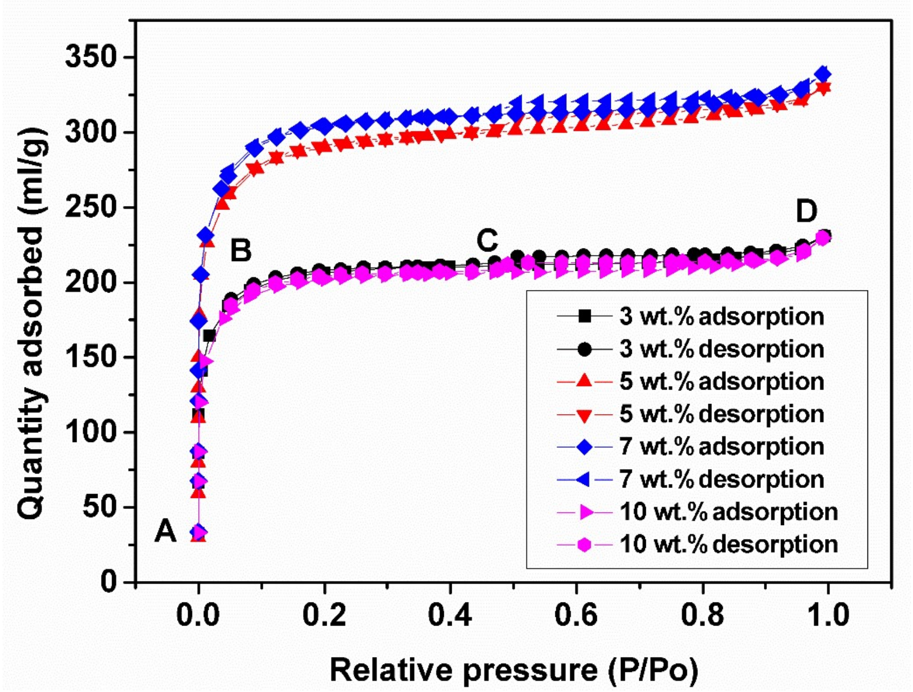

Figure S4. The Isotherm linear plots of AC/graphene/PVDF electrode materials with different graphene conductive additive amounts of 3, 5, 7 and 10 wt %.

The Isotherm linear plots of AC/graphene/PVDF electrodes in Figure S6 show these electrode materials have IV type [1-2]. When N_2 was absorbed in pores of electrode materials, monomolecular adsorption layer is formed first, corresponding to AB segment. And then, multimolecular adsorption layer happens, represented by CB segment. With the increase of relative pressure, the capillary condensation takes place, related to CD segment, whose flat increase reflects the wide pore size distribution of these electrode materials. When all pores are filled by N_2, the curve will trend to flat, denoting absorption saturation, which stage occur at relative pressure up to 1.

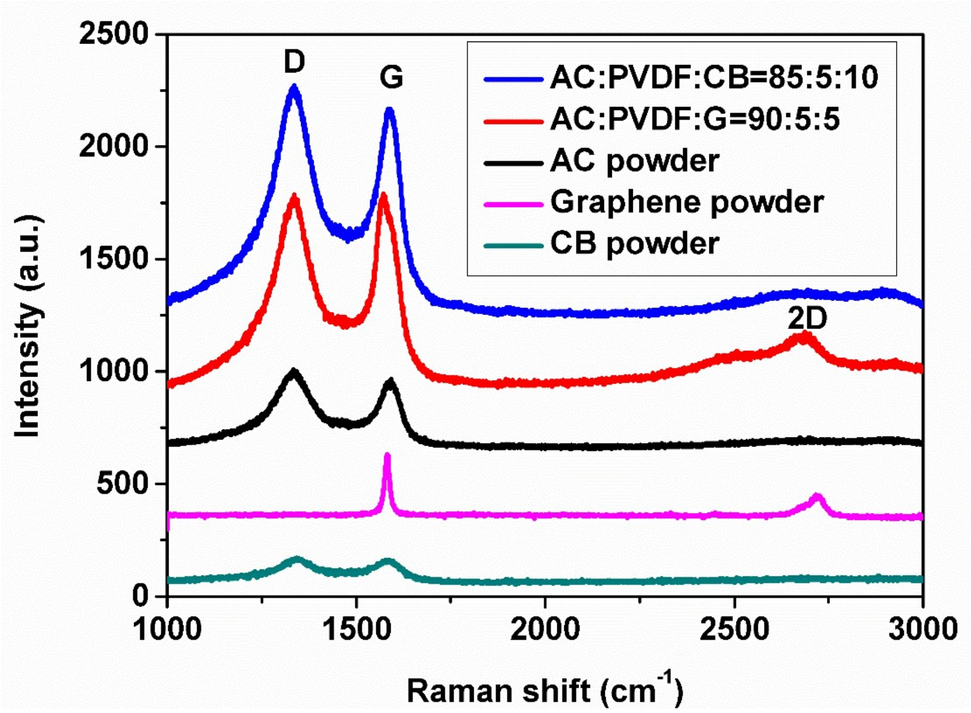

Figure S5. The Raman spectra of raw materials (AC, high quality few-layer graphene and CB) and composite electrode materials (AC@CB and AC@G).

For carbon materials, D band is known as the disorder, defect or sp^3 diamond band, and G band is known as the sp^2 graphite or tangential band. 2D band is commonly seen in 2D graphene, which is an important feature to distinguish graphene from bulk graphite. I_D/I_G is related to the degree of graphitization, and the smaller ratio means the higher graphitization degree [3-4]. As shown in Fig. S8, the Raman spectra of AC and CB just have D band and G band (D bands around 1336 and 1342 cm^{-1} and G bands around 1590 and 1582 cm^{-1}), and graphene has the G band around 1582 cm^{-1} and 2D band around 2718 cm^{-1}. After the addition of CB as conductive additive, no obvious change of the Raman spectrum is observed. However, with the addition of graphene, I_D/I_G reduces, indicating the AC@G composite electrode with higher graphitization degree. Meanwhile, 2D band occurs, denoting the existence of graphene.

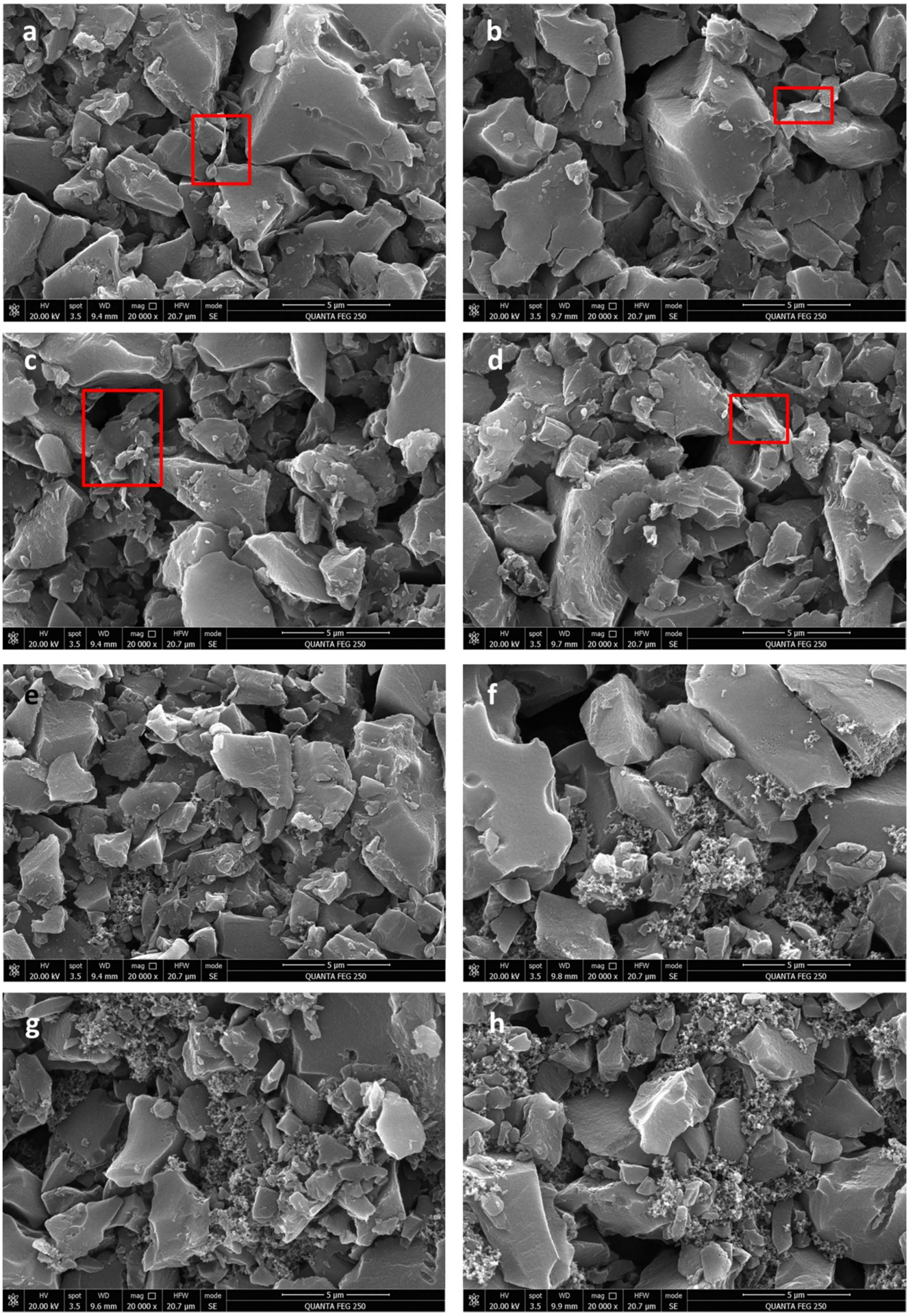

Figure S6. The SEM images of AC@G@NiF/G electrodes with graphene conductive agent content of (a) 3 wt %, (b) 5 wt %, (c) 7wt %, (d) 10 wt % and AC@CB@NiF/G electrodes with CB content of (e) 3 wt %, (f) 5 wt %, (g) 7 wt %, (h) 10 wt %. The red rectangles in a, b, c and identify typical graphene sheets in composites.

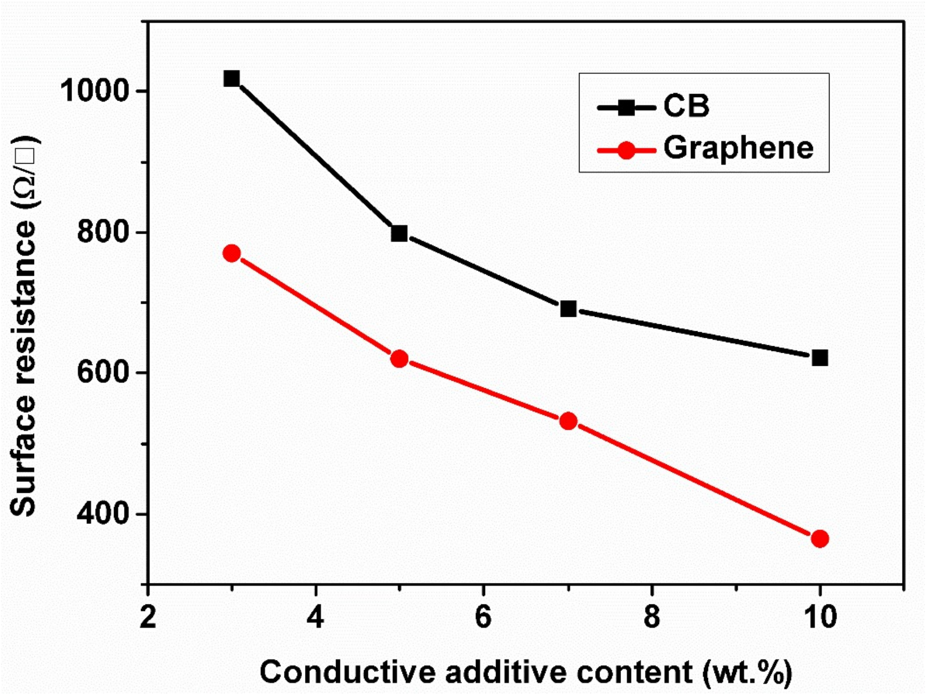

Figure S7. The surface resistance of AC composite materials with different content of graphene and CB conductive additives.

AC composite slurries were prepared for electrodes with graphene and CB as conductive additives, respectively (the content of conductive additive is from 3 to 10 wt %). Afterwards, slurries were blade coated on insulating polyethylene terephthalate (PET) plastics films which avoid the interference from conducting substrates to the subsequent surface resistance of electrode materials, and then dried at 120 °C in vacuum overnight. The thickness of the composite material films were 34 ± 2 μm. The measured surface resistance demonstrates that few layer graphene as conductive additive directly improves the conductivity of the electrodes and is more effective than CB.

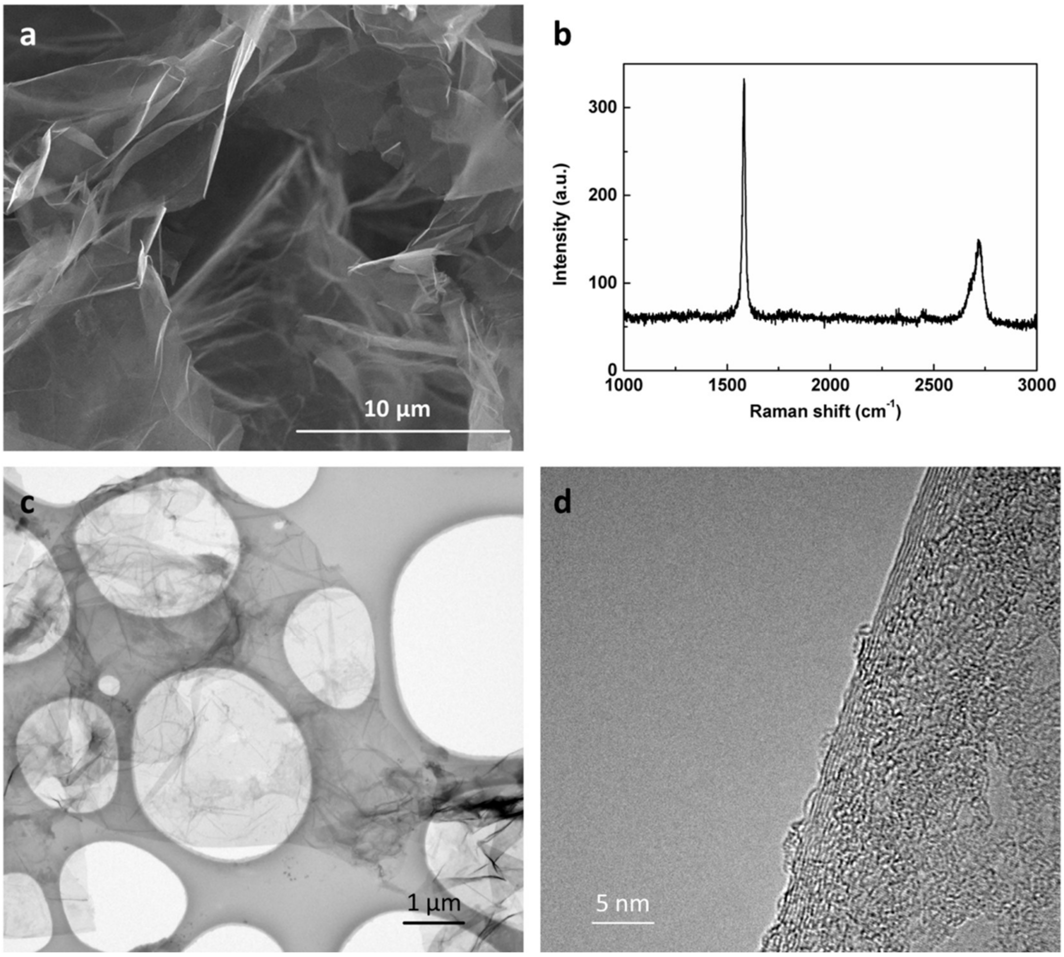

Figure S8. The characterizations of high quality few-layer graphene. (**a**) SEM image (scale bar = 10μm), (**b**) Raman spectrum, (c) TEM image (scale bar = 1μm) and (d) HRTEM image (scale bar = 5 nm).

From Figure S8a,c, graphene with large lateral size and undamaged structure can be observed. The absence of D peak (~1369 cm^{-1}) in Raman spectrum in Figure S8b means low defeat level of the high quality graphene. According to the HRTEM image in Figure S8d, the graphene is counted about 8 layers. It also can be prove by the I_G/I_{2D} value larger than 0.8 from Raman spectrum, indicating few layer graphene [3]. So the layer of graphene is deduced 5-10 layers.

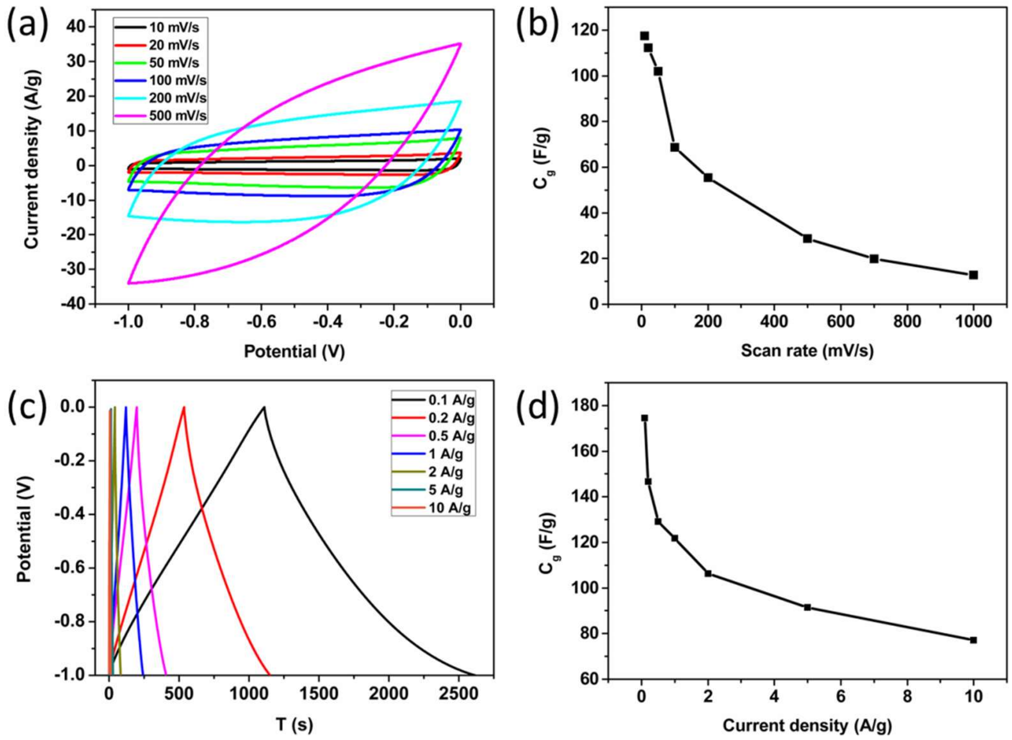

Figure S9. The electrochemical properties of the AC@G@NiF/G-5 electrode. (**a**) CV curves at scan rate various from 10–500 mV/s and (**b**) the corresponding mass specific capacitances as a function of scan rates. (**c**) GCD curves at different current densities of 0.1–10 A/g and (**d**) the corresponding mass specific capacitances as a function of current densities.

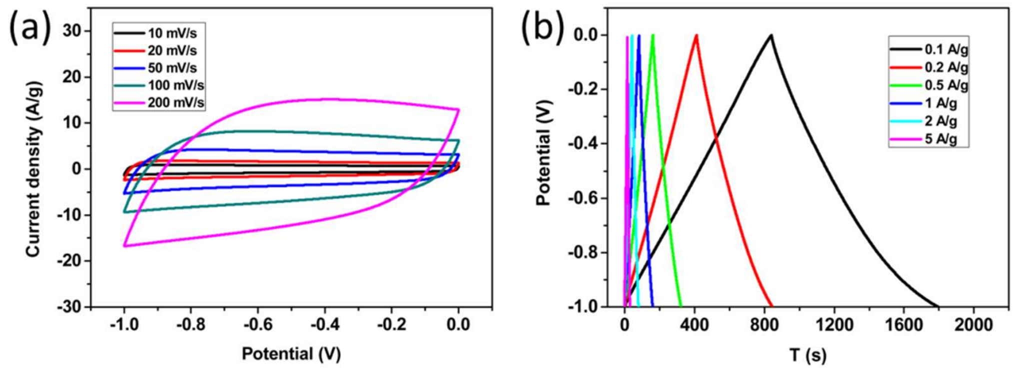

Figure S10. (**a**) CV curves at scan rate various from 10 to 200 mV/s and (**b**) GCD curves at different current densities from 0.1 to 5 A/g of the AC@CB@NiF/G-10 electrode.

References

1. Barrett, E.P.; Joyner, L.G.; Halenda, P.P. The determination of pore volume and area distributions in porous substances. I. Computations from nitrogen isotherms. *J. Am. Chem. Soc*. **1951**, *73*, 373-380, doi:10.1021/ja01145a126.
2. Sing, K.S.W.; Everett, D.H.; Haul, R.A.W.; Moscou, L.; Pierottp, R.A.; Rouquerol, J.; Siemieniewska, T. Reporting physisorption data for gas/solid system with special reference to the determination of surface area and porosity. *Pure Appl. Chem.* **1985**, *57*, 603–619.
3. Bhaviripudi, S.; Jia, X.; Dresselhaus, M.S.; Kong, J. Role of kinetic factors in chemical vapor deposition synthesis of uniform large area graphene using copper catalyst. *Nano Lett.* **2010**, *10*, 4128-4133, doi:10.1021/nl102355e.
4. Roh, J.S. Structural Study of the Activated Carbon Fiber using Laser Raman Spectroscopy. *Carbon Lett.* **2008**, *9*, 127-130.
